# Unifying work values: establishing a circular framework based on basic human values

**DOI:** 10.3389/fpsyg.2025.1526799

**Published:** 2025-08-11

**Authors:** Jannick Schneider, Marcel Kern, Timo Lorenz

**Affiliations:** ^1^Fraunhofer Institute for Industrial Engineering, Stuttgart, Germany; ^2^Faculty of Psychology, Ruhr University Bochum, Bochum, Germany; ^3^Department of Psychology, Medical School Berlin, Berlin, Germany

**Keywords:** work value, basic human value, neoliberalism, person-organization fit (PO fit), organizational psychology, organizational culture

## Abstract

This paper addresses challenges in personal work value research, particularly the lack of theoretical and explanatory foundations. With a focus on lists of constructs and potential biases and blind spots in past work value conceptualizations, the diversity of instruments used to assess values in organizational settings has led to ambiguity and incomplete progress in the field. By integrating propositions from basic value research, this paper develops a comprehensive work value theory. The theory is based on the compatibility and conflict of underlying basic motivational goals in work contexts, as postulated by the theory of basic human values. We review past instruments from work value research to consider a broad range of constructs with the purpose of refining broader work value constructs and enhance their theoretical capabilities in organizational settings. To achieve that, we resolve definitional inconsistencies, enable a context-sensitive theorizing of values in a motivational circumplex and broaden the scope of work value constructs to cover personal and social-focused dimensions. The latter is discussed considering the fantasmatic logic of neoliberal ideology. The developed theoretical framework can guide future research on the role of work values for organizational behavior and organizational performance, as well as on the role of fit between personal and organizational values. The paper concludes by highlighting the need to empirically validate the proposed work value model across different cultures and organizational contexts.

## Introduction

In psychological research, individuals’ work values have been assessed using ranking scales, preference ratings, decision making in ill-defined problems for identifying behavioral guiding principles and natural language processing, as well as qualitative approaches like interviews, observations and archival data (Hattrup et al., 2007; Ponizovskiy et al., 2020; Ratchford et al., 2023; Wright et al., 2017). Potentially due to its low-threshold application, validation, and statistical properties, the most prevalent assessments are rating scales (Schwartz, 1994), where respondents rate various work aspects and outcomes according to their subjective importance (Lyons et al., 2010).

The Integrated Work Value Scale (IWVS; Busque-Carrier et al., 2022a) unites work value items based on past instruments into a single questionnaire. Thus, it can be seen as the most comprehensive instrument to assess work values to date. While their approach offers valuable insights into the landscape of work value constructs, the questionnaire and work value conceptualization lacks an elaborated, unified theoretical framework. Hence, theoretical conceptualizations of the internal structure of work values should be considered, opposed to the past focus on lists of constructs (Arieli et al., 2020b; Borg et al., 2019; Lyons et al., 2010). Additionally, the aggregation of questionnaires could replicate blind spots from past research which potentially restricts theoretical comprehensiveness. Moors et al. (2017) identified a potential gender bias in work value conceptualization due to the underdevelopment of items addressing social aspects of work.

In other studies, the integration of disparate constructs results in unclear measurements and inferences of independent and potentially redundant or biased work value lists. Blending values with career anchors, career and work orientations, motivation and needs impedes theoretical inferences exclusively attributable to employees’ work values (Abessolo et al., 2021; Furnham et al., 2021; Stiglbauer et al., 2022). Additionally, long lists of work values contradict theoretical requirements of parsimony (Aguinis and Cronin, 2022). For instance, differentiating constructs like pay, perks, benefits, and bonuses within a single scale may indicate redundancy and a lack of distinctiveness in motivational foundations (Furnham et al., 2021).

Why does a clear theoretical conceptualization and assessment of work values matter? Values are an integral part of organizations’ and employees’ identities (Arieli et al., 2020b). For instance, the knowledge of employee or team values can be a powerful tool for leaders to react to or to create change in complex, fast changing environments (Van Dick et al., 2018). Therefore, values are significantly associated with outcomes such as creativity, proactive behavior, reactions to change, and wellbeing (Anglim et al., 2022; Arieli et al., 2020a, 2020b; Bojanowska et al., 2022). Furthermore, several theories in work and organizational psychology are based on the concept of values. Theories on, for example, leadership (transformational leadership, identity leadership; Bass and Steidlmeier, 1999; Haslam et al., 2022), person-organization fit (PO-Fit; Kristof-Brown et al., 2023), organizational culture (Schneider et al., 1995), work motivation (social identity theory, personality theory of motivation; Ellemers et al., 2004; Locke and Latham, 2004), wellbeing (self-affirmation theory; Rader et al., 2024), team performance (Parks-Leduc et al., 2024) and ethical decision making (contingency model of ethical decision making; Fritzsche and Oz, 2007) use personal values as a fundament to explain organizational behavior. Hence, values are important for our theories in organizational settings, but their theorizing is untapped and potentially biased when it comes to work values. This paper aims to rethink and build upon existing constructs and conceptualizations of work values to develop a unifying theoretical foundation.

We propose to transfer the cross-culturally validated tenets of basic human value research to the work context to obtain a unifying work value circumplex that illustrates the compatibility and conflict of underlying goals and motivation in work contexts. Hence, this paper contributes to the literature in four ways: (1) by formulating clear-cut definitions of work values to resolve construct proliferation; (2) by developing a theoretical framework to tackle the use of lists and guide future research with contextual sensitivity (Flake et al., 2017) and to benefit diagnostic purposes in work settings (e.g., Moldzio et al., 2021; Sackett et al., 2022); (3) by illuminating blind spots and biases in previous work value instruments through paralleling past work value research to neoliberal ideology; and (4) by corroborating the circumplex structure of work values through additional theorizing to align work values with basic value literature.

In the following, we will begin with defining employees’ work values and their distinction to related constructs, explaining why these differentiations in the context of work and organizational psychology matter. Then, our basic propositions underlying the theoretical advancements will be discussed. Finally, we present the theoretical framework with supporting theoretical arguments from related disciplines, as well as implications for research in organizational and work settings.

## Defining the construct

The conceptualization of personal work values in past research underlies heterogeneity and suffers from construct proliferation (Dose, 1997; Kis et al., 2025; Shaffer et al., 2016). Different studies describe the interchangeability of values, motives and needs due to similar measurement approaches (Pincus, 2024; Stiglbauer et” al., 2022; Knardahl and Christensen, 2024). Others view them as distinct (Sagiv and Schwartz, 2022) but related constructs (Busque-Carrier et al., 2022a) where values are cognitive representations of motives (Kooij et al., 2011; Rokeach, 1973). Some studies define them as affective constructs associated with positive feelings (Gessnitzer et al., 2015) contrasting a strict cognitive focus (Borg et al., 2019). Additionally, differentiations in the behavioral activation and inhibition system (approach and avoidance motivation) led to the study of ideal and counter-ideal values (Schuh et al., 2018; Van Quaquebeke et al., 2014).

Blending work values with other constructs like work orientations (Stiglbauer et al., 2022) or career anchors (Abessolo et al., 2021) confounds theoretical and empirical advancement in the study of values in organizations (Borg et al., 2019). Given the scattered empirical and theoretical perspectives on values, our aim in the following is to develop a clear representation of the underlying construct.

### The conceptualization of work values and its theoretical relevance

Work represents one domain of individuals’ lives where work values are a contextualization of and derived from basic values (George and Jones, 1997; Ros et al., 1999). Basic values as context-free constructs pertaining multiple life domains were extensively differentiated from related constructs like motives, needs, traits, and attitudes (Arieli et al., 2020b; Borg et al., 2019; Sagiv and Roccas, 2021). Hence, to address construct proliferation and the heterogeneity of past research on work values, our conceptualization is based on the central assumptions of the basic values definition (Sagiv and Schwartz, 2022). We build our arguments on the roots of value literature (Kluckhohn, 1951; Rokeach, 1973; Schwartz, 1992), comprehensively researched and contemporary frameworks (Sagiv and Schwartz, 2022) and their extensions to work contexts (Arieli et al., 2020b; Maio et al., 2020).

Table 1 gives an overview of the definitional components of work values with exemplifying relations to their nomological network in work contexts to underline why these components matter (Sagiv and Schwartz, 2022, p. 520). Additional differentiations from vocational interests and work orientations are important (Arieli et al., 2020b). Vocational interests as defined by the RIASEC model (Holland, 1997) are focused on specific tasks and activities at work and do not represent overarching guiding principles (Arieli et al., 2020b; Liu et al., 2024; Sodano, 2011). Concerning work orientations, work values are associated with the way in which people think about work (Rosso et al., 2010). The different forms of job, career, and calling orientations are associated with different levels of emphasis on interests of others or self-interests in personal value structures (Arieli et al., 2020b).

**Table 1:** Definitional components and exemplifying relations of work values to nomological network.

Definitional component	Nomological network
Individuals perceive their “own values as inherently desirable, worthy and good.” Thus, work values reflect what is perceived as important to people at work. Individuals want to act in ways that allow them to promote their work values and attain the underlying goals.	Conceptualization of eudaimonic wellbeing (Bojanowska et al., 2022; Waterman et al., 2008) and value-based behavior to enhance wellbeing (Sheldon and Krieger, 2014) Conceptualization of meaning of work (England, 1967; Lysova et al., 2019; Rosso et al., 2010; Shea-Van Fossen and Vredenburgh, 2014)
Due to the social desirability of work values, they can be utilized to work toward a common goal with others.	Social identity theory (Tajfel and Turner, 1979) Organizational identification (Weisman et al., 2023), person-organization fit (Kristof-Brown et al., 2023; Van Vianen, 2018) and identity leadership (Haslam et al., 2022)
Work values are hierarchically ordered. Thus, the extent to which specific work values motivate actions depends on the subjective work value hierarchy and relative importance.	Value-based interventions and potentially even voluntary value change through changing the hierarchy of important beliefs (Russo et al., 2022)
Work values as cognitive representations of basic motivations and broad goals are more easily accessible for individuals and can be used to reflect and communicate about them, and consciously direct behaviors in specific situations.	Self-affirmation theory (Rader et al., 2024; Sherman and Cohen, 2006): Conscious access to work values enables individuals to secure mental resources and cope with stressful situations at work when reflecting on their important guiding principles (Russo-Netzer and Atad, 2024).
Personal work values form a sense-making system which is used as standards to evaluate and justify choices of oneself and others.	Explaining intergroup and intragroup conflict, when team behaviors oppose individuals work values (Krueger et al., 2022).

### A unified definition of work values

Considering the definitional components outlined in Table 1, we base our theoretical framing of work values on the following conceptualization (Arieli et al., 2020b; Borg et al., 2019; De Clercq et al., 2008; Lyons et al., 2010; Maio et al., 2020). Work values describe the specific expression of basic values at work (Elizur and Sagie, 1999; George and Jones, 1997; Ros et al., 1999). They are cognitive representations of basic motivations as desirable work contexts and goals that serve as guiding principles in people’s working life. Hierarchically ordered, they describe expectations and preferences at work according to their relative importance (Arieli et al., 2020b; Lyons et al., 2010; Sagiv and Schwartz, 2022). Consequently, behavioral choices and outcomes at work are evaluated as more or less desirable (Dose, 1997; Kluckhohn, 1951; Rokeach, 1973; Schwartz, 1994). As a results, these generalized beliefs and broad goals influence decision making and action of individuals and other social units through their varying desirability and importance of work aspects and outcomes (Sagiv and Roccas, 2021). Utilizing this definition, researchers can build a foundation for future work value studies to address issues of construct proliferation. In the following, we present our theoretical advancements grounded in this definition.

## Work values—integrating theoretical perspectives

The use of lists and potential biases in work value conceptualizations as presented above can be tackled by referring to a growing body of research that provides empirical evidence for integrating work values in the theory of basic human values (Schwartz, 1992). The theory established a widely used and empirically supported theoretical framework in over 80 countries (Sagiv and Schwartz, 2022) with elaborated processes on how fundamental personal values can affect behavior (Sagiv and Roccas, 2021). Basic values are considered in dynamic relations of compatibility and conflict based on a fundamental motivational continuum (Borg et al., 2019; Elizur and Sagie, 1999; Schwartz, 1992). The theory and its circular structure provides consistent and cross-culturally generalizable evidence on associations with religiosity, altruistic and anti-social behavior (aggression, unethical and delinquent behavior), and political activism, ideology and voting choice (Sagiv and Schwartz, 2022). The content of these basic values transcends different life domains as trans-situational goals. Nevertheless, their absolute level of importance can vary across contexts (Daniel et al., 2012b) depending on which roles individuals are assigned to or assume (e.g., family member, student, employee; Daniel et al., 2012a). Thus, individuals differentiate in their value priorities across life domains.

Focusing on the relevant domain for our theorizing, individuals’ working life in organizations, this circular structure and the theoretical assumptions of employees guiding principles were replicated as well (Arieli et al., 2020b). Hence, as organizations are a further step of individuals socialization (e.g. Den Boer et al., 2024), it is important to consider employees work values in relation to the expressed values of an organizations’ culture (Arieli et al., 2020b; Kristof, 1996). Integrating these two perspectives, researchers aligned factors of organizational culture with propositions of the TBHV (e.g., Organizational Culture Profile, OCP; Borg et al., 2011; De Clercq et al., 2008), supporting its applicability in working contexts. Additionally, studies assessing a broad range of work value items display considerable alignment with the circularity-assumptions of conflict and compatibility (Albrecht et al., 2020; Borg et al., 2019; Schneider et al., 2024).

### The theory of basic human values in work contexts

As conscious motivational goals, basic values respond to three universal requirements of human existence that all individuals and societies must address (Schwartz, 1992). From an evolutionary perspective, these constitute needs of individuals as biological organisms, requisites of coordinated interaction, and survival and welfare needs of groups. Two pairs of higher-order basic value dimensions represent the motivational continuum with value constructs arranged according to their compatibility and conflict of underlying goals. *Self-Transcendence* vs. *Self-Enhancement* and *Openness to Change* vs. *Conservation* are often transferred to work context as *Social*, *Prestige*, *Intrinsic* and *Extrinsic* dimensions (e.g., Busque-Carrier et al., 2022a; Ros et al., 1999). *Social-*related work values reflect the importance of positive social relationships and the possibility to contribute to society. *Prestige*-related work values represent goals regarding power, authority, influence, and success at work. Importance of autonomy, interest, enjoyment, and creativity are expressions of *Intrinsic-*related work values. In contrast, *Extrinsic-*related work values relate to the importance of job security and maintaining order in an employee’s life.^1^ The values are arranged in a circular format based on the compatibility or conflict between their underlying basic motivational goals. Consequently, values that represent conflicting goals are positioned further apart, whereas those with compatible goals are adjacent. This arrangement suggests that compatible values tend to foster similar perceptions, preferences, and behaviors, as their underlying goals are more likely to be pursued through similar actions. In contrast, conflicting values hinder the simultaneous pursuit of their respective goals, as advancing one goal can obstruct another (Maio et al., 2009; Schwartz, 1992, 2021).

**Figure 1:**
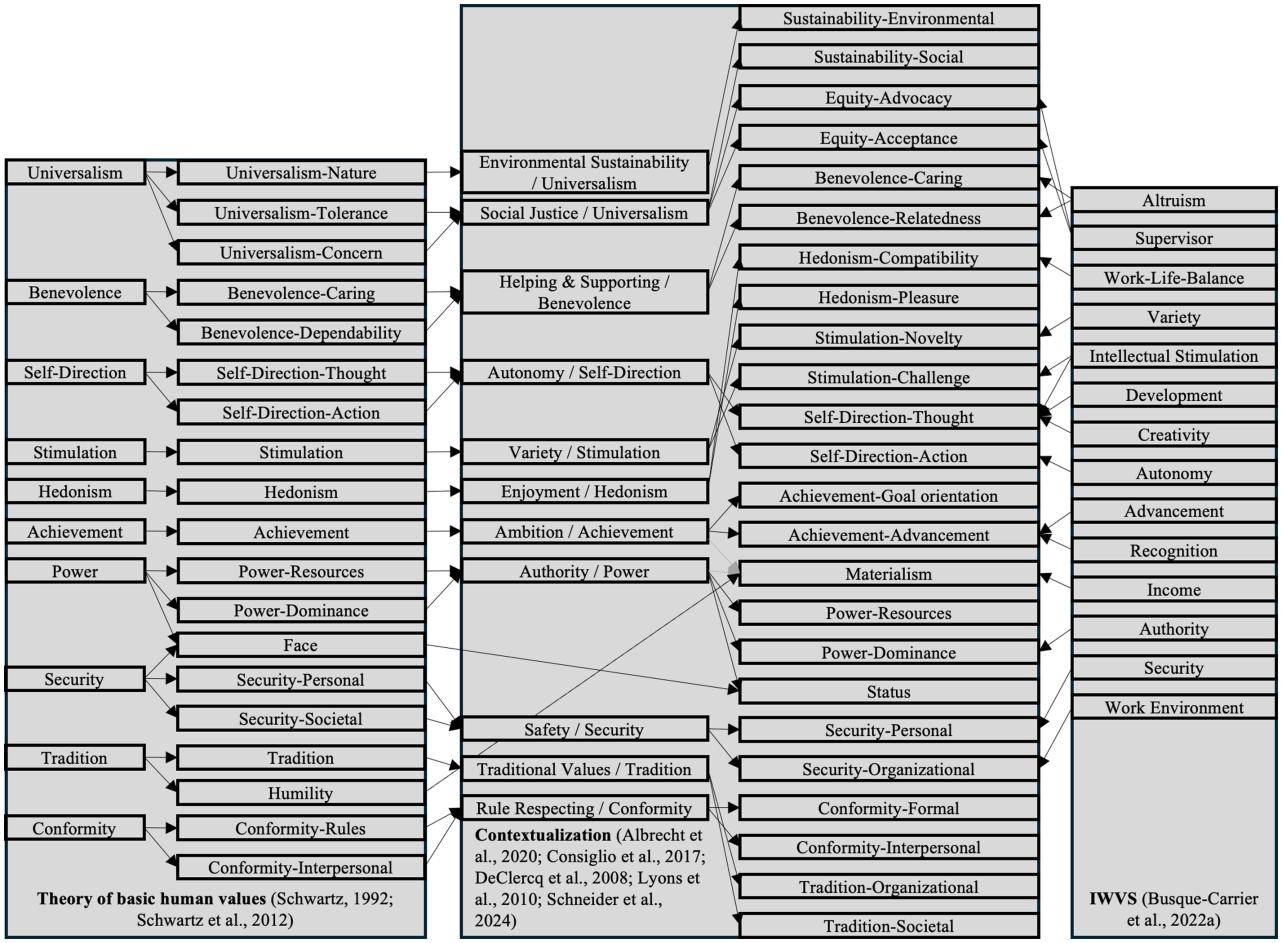
Theoretical model of the proposed work value circumplex and motivational continuum.

According to the theory of basic human values conflict and compatibility are derived from values which express a personal (mainly *Self-Enhancement* and *Openness to Change*) or social (mainly *Self-Transcendence* and *Conservation*) focus. Furthermore, differentiations regarding Growth - Anxiety free (*Self-Transcendence* and *Openness to Change*) and Self-Protection—Anxiety avoidance (*Conservation* and *Self-Enhancement*) values can be made (Sagiv and Schwartz, 2022). Those aspects of conflict and compatibility manifest in 10 broad basic value constructs (*Self-Direction, Stimulation, Hedonism, Achievement, Power, Security, Tradition, Conformity, Universalism, Benevolence*).

Research has successfully incorporated work value items and constructs into the basic values of Schwartz (1992). Considering two examples, De Clercq et al. (2008) classified over 1,500 work value items into the value conceptualizations of the TBHV. Additionally, Borg et al. (2011) linked the Organizational Culture Profile (O’Reilly et al., 1991) to Schwartz’ value constructs. Therefore, these universal value constructs tend to be appropriate to integrate findings from organizational research (Arieli et al., 2020b). Research has validated instruments assessing 11 broader work value constructs replicating the value circumplex of Schwartz (1992) in various cultural contexts for work settings (Albrecht et al., 2020; Consiglio et al., 2017; Schneider et al., 2024). They all depicted considerable alignment with the theoretical propositions of the theory of basic human values. However, Schwartz et al. (2012) argued that there is substantial heterogeneity in the broader 10 value constructs (Beierlein et al., 2012). This led to the development of a refined theory of basic human values with revised definitions (Schwartz and Cieciuch, 2022). How these developments can be transferred to the realm of work, is discussed next.

### Theoretical refinements in work contexts

Specific value constructs tend to be more appropriate for organizational research (Bagozzi and Edwards, 1998; Salgado, 2017; Stephan, 2020) and they increase predictive validity and practical relevance for theorizing (Schwartz et al., 2012). De Clercq et al. (2008) found additional work value items, which could not be theoretically assigned to one of the 10 basic value constructs. This supports the need for further evaluation of theoretical soundness of the broader basic value constructs in work contexts (Arieli et al., 2020b; Maio et al., 2020). The refinement for work contexts enhances not only theoretical comprehensiveness. We ultimately aim at integrating past approaches of contextualizing the theory of basic human values in work contexts (Albrecht et al., 2020; Consiglio et al., 2017; Schneider et al., 2024) with other comprehensive approaches in work value research (Busque-Carrier et al., 2022a). In the following, we will first discuss the content of our work value theory and how these differentiations potentially relate to behavior in organizations; second, we will address the underlying structure of work values; and third, we will provide supporting theoretical arguments.

### The content of the circular work value (CWVT)

Figure 1 includes the deduction of our work value constructs integrating past research findings. Our approach aimed at integrating four overlapping research streams: (1) We used the refinement of the basic value circumplex as a theoretical starting point (Schwartz and Cieciuch, 2022) to build on contemporary personal value literature from a cross-cultural perspective (Sagiv and Schwartz, 2022), (2) we utilized past discussions of the TBHV in work settings and how contextual specificities at work are important to consider (Arieli et al., 2020b; De Clercq et al., 2008; Lyons et al., 2010; Maio et al., 2020), (3) we integrated contextualized assessments of the 10 basic human values at work (Albrecht et al., 2020; Consiglio et al., 2017; Schneider et al., 2024), and (4) we build on the work of Busque-Carrier et al. (2022a) on reviewing a broad range of previous work value models. The development process resulted in the construct differentiations as elaborated next and defined in Table 2.

**Figure 2:**
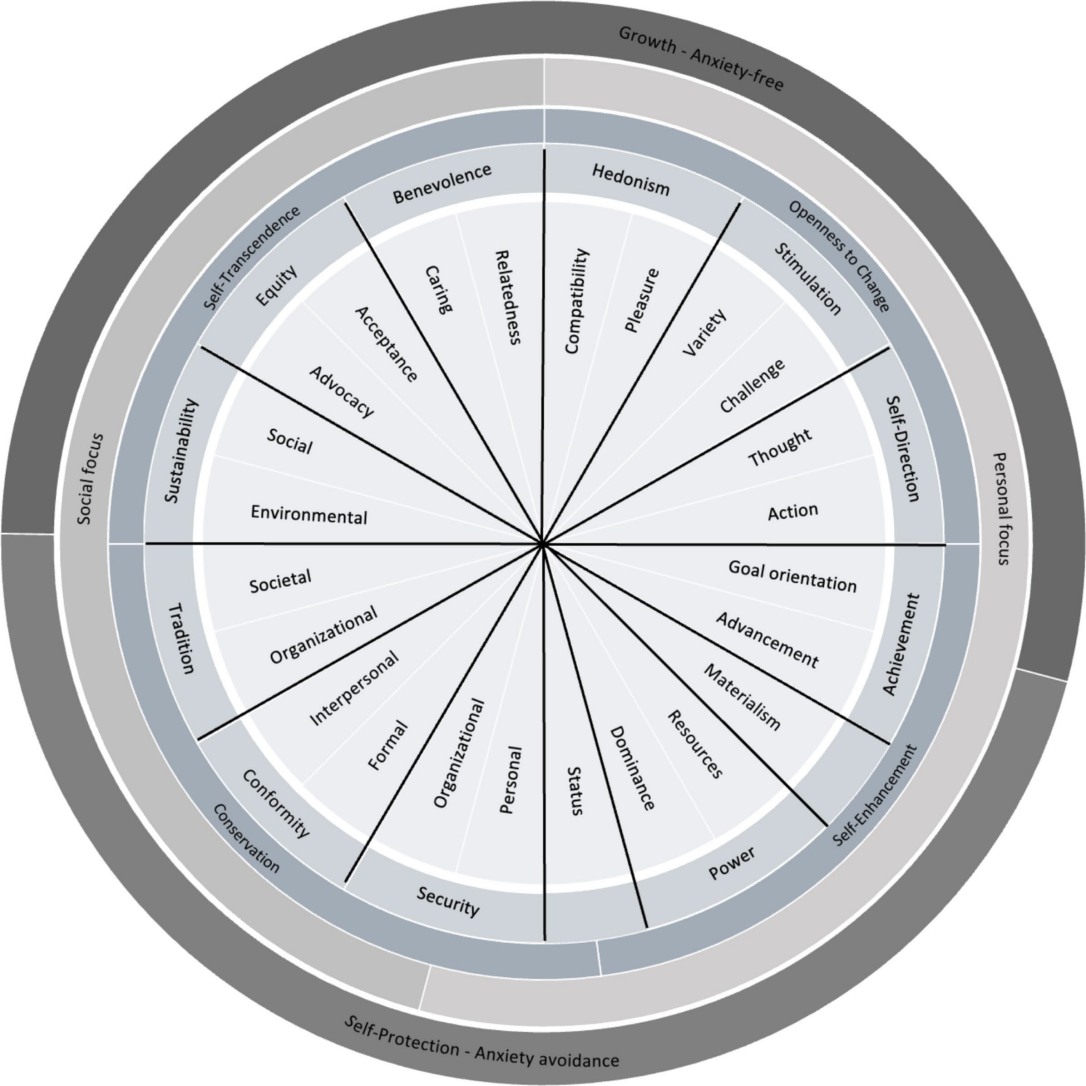
Flow chart for the advancement and contextualization of the theory of basic human values in relation to the IWVS.

**Table 2:** Definitions of proposed work values.

Higher-order work value	Work value	Definition: importance of…	Work value sub-construct	Definition: importance of…
Self-enhancement	Status	Maintaining one’s social status and prestige at work	Not specified	
	Power	Leadership roles and influence through control or dominance over people and resources	Dominance	Power through control over people at work
			Resources	Power through control over resources at work
	Materialism	Material goods, wealth and luxury achieved through work	Not specified	
	Achievement	Personal success at work as defined by recognition of one’s abilities and products in the organization	Advancement	Recognition of own skills and work results in accordance with social standards in the organization
			Goal orientation	Pursue a goal through one’s own work performance and demonstrate initiative and competence
Openness to change	Self-direction	Independent thought and decision-making, creating, and exploring at work; freedom to choose how to perform one’s job	Action	Freedom in determining one’s own actions at work
			Thought	Freedom to cultivate one’s own ideas and abilities at work
	Stimulation	Variety, novelty, and challenges in work situations and contexts	Challenge	Challenges and adapting to changing circumstances at work
			Variety	Variety and novelty at work
	Hedonism	Pleasure in doing work, compatibility between work and one’s recreational and leisure interests	Pleasure	Pleasure and sensuous gratification for oneself at work
			Compatibility	Compatibility of work and recreational and leisure interests
Self-transcendence	Benevolence	Devoting oneself to the needs of people with whom one is in frequent work contact and creating harmonious and supportive work relationships	Relatedness	Harmonious working relationships characterized by reliability and trustworthiness
			Caring	Considering the needs and promoting the wellbeing of other people at work
	Equity	Fairness, respect, protection against discrimination for all members of the work organization	Acceptance	Acceptance and understanding of the individual differences of all members of the organization
			Advocacy	Commitment to justice, equality and protection against discrimination for all members of the organization
	Sustainability	Contributing to the welfare of society through work; socially responsible policies	Social	Contributing to a fair and equal society through work
			Environmental	Preservation of the natural environment at work
Conservation	Tradition	Respect, acceptance, and diffusion of traditions, culture, and customs at work	Societal	Maintaining and preserving societal, religious and family traditions, culture and customs at work
			Organizational	Maintaining and preserving organizational traditions, culture and customs
	Conformity	Complying and adapting to management expectations and norms, sacrificing personal inclinations to preserve organizational order	Interpersonal	Conformity with the norms and expectations of others at work
			Formal	Conformity with guidelines and rules at work
	Security	Safety, stability, avoiding risks in the work and organizational setting	Organizational	Safety in the organizational environment
			Personal	Safety in one’s job and professional future

Work value definitions are derived from Albrecht et al. (2020), Consiglio et al. (2017), Sagiv and Schwartz (2022), and Schneider et al. (2024).

### Self-transcendence

#### Benevolence

The construct of *Helping and Supporting* by Albrecht et al. (2020) represents the contextualization of *Benevolence*. As the definition in Table 2 shows, two possible facets can be “devoting oneself to the needs of people with whom one is in frequent work contact” and “creating harmonious and supportive work relationships.” Schwartz et al. (2012) differentiated *Benevolence* into *Dependability* (“being a reliable and trustworthy member of the ingroup”) and *Caring* (“devotion to the welfare of ingroup members”). De Clercq et al. (2008) suggested an additional value to cover work value items named *Relatedness* (“motivation to form good relationships with others in the workplace”), which can further be found in the work value definition by Consiglio et al. (2017) (see Table 2). *Benevolence-Caring* matches conceptually to the *Altruism* (“help others at work/promote their wellbeing”) work value as defined by Busque-Carrier et al. (2022a). These findings support a differentiation of our work value *Benevolence* into *Relatedness* and *Caring* (see Table 2).

#### Equity

Compared to the ingroup focus of *Benevolence, Universalism* aims for a broader social environment with tolerance and justice “for all people” (Schwartz and Cieciuch, 2022). *Universalism*-*Tolerance* (“acceptance and understanding of those who are different from oneself”) and *Concern* (“Commitment to equality, justice, and protection for all people”) are diversifications made in the refined theory of basic human values (Schwartz et al., 2012; Schwartz and Cieciuch, 2022). The definition proposed by Consiglio et al. (2017) emphasizes the importance of fairness and respect toward members of the work organization, along with the implementation of “socially responsible policies” that extend beyond the boundaries of the organization (see Table 2). In contrast, the items developed by Albrecht et al. (2020) expressed the goal of *Social Justice* as contributing to broader society through one’s work (“to make the world a better place”) leaving out the internal organizational perspective. The questionnaire of Schneider et al. (2024) assesses work value items which express both perspectives.

Furthermore, Busque-Carrier et al. (2022a) corroborate this organizational perspective with their work value for fair and equal treatment by supervisors. Based on this, we differentiated the work value of *Equity* into the sub dimensions of *Advocacy* and *Acceptance*. The work values state the importance of goals addressing fairness and justice of people at work (*Advocacy*) and the respect and acceptance of individual differences of people whom one encounters at work (*Acceptance*).

#### Sustainability

Schneider et al. (2024) argued for differentiations in *Social Justice* addressing the target group (organizational vs. societal). These are comparable to the different foci of Albrecht et al. (2020) for *Social Justice* in the society and Consiglio et al. (2017) for *Universalism* in the organization (as illustrated above). In line with theoretical contributions made by Lyons et al. (2010) work values may represent goals and expectations targeting the individual, the job/organization, or the society. As concluded earlier, *Equity* and *Benevolence* pertain to the organizational/job level. To acknowledge the societal focus of Lyons et al. (2010) and the conceptualization of Albrecht et al. (2020), we formulate another broader *Sustainability* work value which exceeds organizational borders in its motivational goals. This work value is differentiated in *Sustainability-Social* and *Sustainability-Environmental* to address the increasing importance of Corporate Social Responsibility (CSR) (Albrecht et al., 2020; Consiglio et al., 2017; Kristof-Brown et al., 2023). Accordingly, “contributing to society” represented by *Social*-related work values in the past becomes apparent (Ros et al., 1999). De Clercq et al. (2008) specified this with the proposition of a *Social Commitment* value (“welfare of all people”).

The definition of *Sustainability-Social* in Table 2 is based on the CSR dimension of people-society. While *Equity* represents the narrower group of people-organization (Paruzel et al., 2021) *Sustainability-Social* includes the conception of *Social Justice* given by Albrecht et al. (2020) for contributing to the broader society. Additionally, the societal focus of socially responsible policies based on the *Universalism* work value definition by Consiglio et al. (2017) is included.

*Sustainability-Environmental* was separated from *Social Justice* in work contexts by the study of Albrecht et al. (2020). Schwartz et al. (2012) highlight the factor of *Universalism-Nature* which addresses the “preservation of the natural environment.” Therefore, Albrecht et al. (2020) and Schneider et al. (2024) support this work value to be a distinct eleventh broader construct besides *Social Justice*. Here in this study, we address a broader *Sustainability* work value for societal and environmental engagement as goals and guiding principles representing contributions to the greater good (Lyons et al., 2010). This is posited analogous to the *Universalism* value of Schwartz et al. (2012).

#### Behavioral implications: work values of self-transcendence

*Self-Transcendence* work values represent guiding principles related to the wellbeing of others, and thus address needs for coordinated interaction, survival and welfare of groups (Schwartz, 1992). Here, the need for positive interaction and flourishing in teams and organizations is covered. Placing high importance on subordinately work values is associated with altruistic behavior to protect and enhance the welfare of others (Arieli et al., 2020a, 2020b). For example, helping colleagues as altruistic organizational citizenship behavior (OCB) is associated with *Benevolence* (Cohen and Liu, 2011). Especially the *Benevolence-Caring* subdimension is aligned with this definition of altruistic behaviors. Attributing high importance to *Self-Transcendence* work values additionally increases the likelihood of successful cooperation with others (Lönnqvist et al., 2013). Prioritizing harmonious working relationships (*Benevolence-Relatedness*) may be more related to a cooperative conflict resolution of individuals (Arieli et al., 2020a, 2020b).

Furthermore, the importance of *Equity* may relate to inclusive workplace behaviors (Shore et al., 2018). For example, with a shared importance of *Equity-Acceptance* in teams, new members could be included as an insider of the work group and encouraged to retain their uniqueness through the acceptance and support of individual differences (Shore et al., 2011). When explaining the effects of CSR approaches in organizations, a differentiation according to the effects of people-employee (related to *Equity-Advocacy*) or people-society (related to *Sustainability-Social*) can be important to consider for PO-fit approaches (Glavas, 2016). *Sustainability*-*Environmental* represents a relevant work value for future research on green employee behavior, as it may shape pro-environmental attitudes (Katz et al., 2022).

### Openness to change

#### Hedonism

*Hedonism* is referred to as “Pleasure in doing work, compatibility between work and one’s recreational and leisure interests” in work contexts (Consiglio et al., 2017). This definition advances the basic value of *Hedonism* (“Pleasure and sensuous gratification for oneself”; Schwartz et al., 2012) by the aspect of work life balance. Based on the distinction in the definition given by Consiglio et al. (2017) (pleasure in doing work and compatibility between work and leisure interests), Schneider et al. (2024) recommend the differentiation of both sub constructs. Busque-Carrier et al. (2022a) as well, include a *Work-Life-Balance* work value in their final scale. The narrower definitions of *Hedonism-Compatibility* and *Hedonism-Pleasure* are given in Table 2.

#### Stimulation

Schwartz et al. (2012) considered two sub-constructs of *Stimulation*, particularly excitement/novelty and challenge. However, these differentiations were not included in the final refinement, as data did not provide evidence for a separation. In work contexts this differentiation may be more considerable. Schneider et al. (2024) support differentiations in their Var*iety* work value between novelty and challenge at work. Busque-Carrier et al. (2022a) formulate a *Variety* (“given to a job where the work tasks are diversified”) and *Intellectual Stimulation* (“a work in which it is possible to solve new problems, where it is necessary to be alert mentally, and which requires an intellectual effort.”) work value. The latter expresses the notion of challenges, problem solving and adapting to challenging work situations and tasks. Based on the definition of *Intellectual Stimulation* (Busque-Carrier et al., 2022a) and *Self-Direction-Thought* (see below and Table 2) the circular continuum seems quite vague due to the intellectual and mental focus of both constructs. Nevertheless, we want to address the given specificities of intellectual efforts (as given in *Self-Direction-Thought*) and the importance of solving new problems and challenges with the need for adapting to changing work tasks and situations (where challenges must not always be intellectual in nature). Therefore, we propose a differentiation of *Stimulation-Variety* (with importance of diversified and novel work experiences) and *Stimulation-Challenge* (with importance of adapting to changing work situations and challenges).

#### Self-direction

According to Schwartz et al. (2012, p. 666) the two sub-constructs of *Self-Direction* “refer to absolute/intrapersonal competence, not external assessments of performance.” It is differentiated by the sub-constructs of *Thought* (“The freedom to cultivate one’s own ideas and abilities”) and *Action* (“The freedom to determine one’s own actions”; Schwartz et al., 2012; Schwartz and Cieciuch, 2022). Similarly, Schneider et al. (2024) proposed distinctions of autonomously performing one’s tasks and developing things and ideas. This differentiation is also present in the definition given by Consiglio et al. (2017). Therefore, in line with Schwartz et al. (2012) we propose two sub-constructs named *Self-Direction-Thought* and *Self-Direction-Action* (see Table 2). *Thought* includes the development and usage of one’s understanding and intellectual competence and hence the development of one’s ideas and abilities (Schwartz et al., 2012). In line with this, the work values of *Development, Intellectual Stimulation* and *Creativity* postulated by Busque-Carrier et al. (2022a) can be included here. Finally, the *Autonomy* work value of Busque-Carrier et al. (2022a) is in line with the *Self-Direction-Action* definition given in Table 2.

#### Behavioral implications: work values of openness to change

The above discussed work values address individuals’ needs as biological organisms for mastery, control, variation to seek an optimal level of activation and the pleasure with satisfying these individual needs (Schwartz, 1992). As discussed by Arieli et al. (2020b), work values related to *Openness to Change* are linked to behavioral outcomes such as autonomy and adaptability to change in organizations. Empirical studies demonstrate positive associations between these work values—particularly *Stimulation–Challenge* and *Self-Direction–Thought*—and both self-reported and expert-rated creative and innovative performance, which are key to driving organizational change (Arieli et al., 2020b; Schwartz et al., 2012).

Moreover, *Openness to Change* work values are positively associated with proactive behaviors, such as initiating change through organizational citizenship behavior (Do Nascimento et al., 2018). However, this relationship may be moderated by factors such as organizational identification (Lipponen et al., 2008) and contextual ambiguity (Grant and Rothbard, 2013). Employees who score high on *Openness to Change* work values also tend to respond more positively to organizational change, although this depends on whether the change is voluntary or imposed (Gonzalez et al., 2023; Sverdlik and Oreg, 2009). Here, prioritizing novelty and variety can be predictive of how employees react to new tasks and responsibilities (as specified by *Stimulation-Variety*). Furthermore, valuing *Hedonism-Compatibility* can be indicative for expectations of flexibility in work arrangements. Here, a work values perspective might contribute additional personal characteristics in research on work-family balance (e.g., Vaziri et al., 2022).

### Self-enhancement

#### Achievement

Schwartz et al. (2012) define the *Achievement* value as “Personal success through demonstrating competence according to social standards.” In contrast to the *Self-Direction* work value, not intrapersonal competence but external assessment according to social standards is dominant in this construct. Correspondingly, Consiglio et al. (2017) define the contextualized work value as “personal success at work defined by recognition […] in the organization.” In line with this conjecture, De Clercq et al. (2008) postulate an additional value in work contexts addressing the striving for admiration and recognition. *Advancement* and *Recognition* are two work values from the work of Busque-Carrier et al. (2022a) which could be included here. The focus lies on the recognition of one’s work in the organization and consequently on advancing one’s position.

De Clercq et al. (2008) postulate an additional value in the dimension of *Self-Enhancement* work values. The work value of *Goal orientation* is defined as the importance of fulfilling “a purpose, show persistence and take initiatives.” As discussed by Schwartz et al. (2012), we as well included the inerpersonal demonstration of competence in this work value definition. Therefore, we differentiate the work value of *Achievement* into the sub-constructs of *Advancement* and *Goal orientation* (see Table 2).

#### Materialism^2^

*Materialism* was additionally identified by De Clercq et al. (2008) to be included in work contexts. As it is defined as “attaching importance to material goods, wealth and luxury,” parallels to the work of Busque-Carrier et al. (2022a) can be drawn. They identified a work value of *Income* addressing financial wealth and a high salary. Schwartz and Cieciuch (2022) assessed a *Power-Resources* factor defined as control of material and social resources with items like “It is important to her to be wealthy” and “It is important to her to own expensive things that show her wealth.” As these items can be considered as indicators for the work value of *Materialism* (De Clercq et al., 2008) we included this construct as a separate work value between *Achievement-Advancement* and *Power-Resources* (considered next).

#### Power

As defined by Schwartz et al. (2012), *Power-Resources* denotes the importance of exerting control over both material and social resources, ultimately contextualizing power at work through resource control. In this vein, emphasis is placed on authorizing and managing resources within the organization. Conversely, *Materialism* underscores the individual’s pursuit of heightened wealth and luxury through their work. Consequently, we distinguish a *Power-Resources* work value, which as well was emphasized by the work of Schneider et al. (2024).

Additionally, as differentiated by Schwartz et al. (2012), a work value of *Power*-*Dominance* can be considered (see Table 2). Here, the control over people represents an additional aspect of *Power* to the above focus on *Resources*. This second sub-factor is corroborated by the constructs assessed by Schneider et al. (2024) for the work value of *Authority*. Busque-Carrier et al. (2022a) as well, derive an *Authority* work value which focusses on the control over the planning, organizing, and carrying out the work of others. Hence, we conclude a *Power-Dominance* work value defined as control over people at work.

Considering empirical and theoretical advancements in basic value research, the value of *Face* (“security and power through maintaining one’s public image and avoiding humiliation”) is important to consider (Schwartz et al., 2012). Moreover, the definition of Consiglio et al. (2017) for the contextualized value of *Power* included definitional components of social status and prestige. Hence, we conclude an additional work value of *Status* for the importance of securing one’s social status and prestige at work (see Table 2).

#### Behavioral implications: work values of self-enhancement

The pursuit of success, control over resources, and the structure of power and status are essential for the functioning of social institutions such as organizations and teams, as well as for individuals to secure the resources and demonstrate competences necessary for continued employability (Schwartz, 1992). In contrast to *Self-Transcendence*, *Self-Enhancement* work values emphasize self-promoting goals over concern for others’ wellbeing (Arieli et al., 2020b). Employees who prioritize work values such as *Status*, *Power*, *Materialism*, and *Achievement* are often more motivated to advance their careers to secure status and prestige. Guiding principles centred on goal orientation, personal success, influence, and the pursuit of wealth and recognition are therefore just as vital for organizational success as the more cooperative, prosocial values associated with *Self-Transcendence* (Arieli et al., 2020b). This may be particularly relevant in organizations or cultures where norms and reward systems are based on merit, competition, and performance, as employees high in *Self-Enhancement* (especially the subdimension of *Achievement-Advancement*) tend to perceive such systems as fair (Fischer and Smith, 2004). From a motivational perspective of Growth–Anxiety-Free values, differentiating *Achievement-Goal Orientation* may help better predict employees’ preferences for demonstrating and experiencing achievement, effort, and competence (Butera et al., 2024), while being less dependent on social recognition of one’s work results.

The differentiation between *Power-Dominance* and *Power-Resources* is important when researchers aim to explain unethical or competitive behaviors in organizations (Sagiv and Roccas, 2021). Individuals who prioritize *Power-Resources* may be more likely to base their decisions on personal benefit rather than on compliance with ethical guidelines, as they prioritize their own advantages over ethical considerations (Arciniega et al., 2019). Additionally, knowledge hiding may occur in individuals who value power through resource control, as they tend to pursue personal gains rather than share expertise, foster collaboration, or contribute to organizational goals (Shen et al., 2025).

Prioritizing control over people at work (*Power-Dominance*) is associated with agentic qualities ascribed to leaders in organizations, such as interpersonal control and social dominance over the opinions and actions of others (Ma et al., 2022). Consequently, this work value may influence behavior and communication within work groups.

### Conservation

#### Security

Safety, stability, and health avoiding risks in the work and organizational setting (Consiglio et al., 2017) is the contextualized definition of the *Security* basic value. In the revised theory of basic human values, Schwartz et al. (2012) differentiate between *Security-Personal* (safety in one’s immediate environment) and *Security-Societal* (safety and stability in the wider society). Albrecht et al. (2020) operationalized their *Safety* work value according to aspects of safety climate (e.g., “To contribute to the safety of colleagues”; “To ensure that danger is minimized”). This view may be too narrow as in work contexts aspects like job security are important to consider (Busque-Carrier et al., 2022a). As the definition of Consiglio et al. (2017) and the work of Schneider et al. (2024) illustrate, safety and stability should as well be considered in direct relation to one’s job and employment.

Consequently, we consider the work values of *Security-Job* (importance of security and stability in one’s job and professional future) and *Security-Organizational* (importance of security and stability in the organizational environment). The first corresponds to Schwartz’ value of *Security-Personal* and the latter to *Security-Societal* with a broader target group. *Security-Organizational* stands in line with the work value definition of Albrecht et al. (2020) and the *Work Environment* value (“working environment is sheltered from bad weather and comfortable”) as defined by Busque-Carrier et al. (2022a). We decided to exclude items which specifically address health promotion/avoiding risks of health impairment, as the position of health in the circumplex tends to show considerable differences, based on the definition of health (Aavik and Dobewall, 2017).

#### Conformity

Originally labeled *Conformity* (Schwartz, 1992), *Rule Respecting* by Albrecht et al. (2020) is defined as the importance of compliance and adaption to management expectations and norms (Consiglio et al., 2017). As differentiated by Schwartz et al. (2012) this compliance can refer to interpersonal norms and expectations or formal rules, laws and obligations. Hence, we introduce this differentiation as well for work contexts with *Conformity-Interpersonal* and *Conformity-Formal*.

#### Tradition

Schwartz et al. (2012) did not differentiate the basic value of *Tradition*, defined as “maintaining and preserving cultural, family or religious traditions.” The focus of the corresponding work value given by Consiglio et al. (2017) lies on respect, acceptance, and diffusion of organizational traditions, culture, and customs. The questionnaire developed by Albrecht et al. (2020) however, focusses on supporting family and societal traditions through one’s work (e.g., “To be able to support the traditions of my society at work”; “To do work which is in keeping with my religious beliefs”). Schneider et al. (2024) included both perspectives in their final model which as well provides evidence for differentiating between two sub-constructs of *Tradition* (*Organizational* and *Societal*; Lyons et al., 2010).

#### Behavioral implications: work values of conservation

These work values reflect the need for coordinated interaction and group welfare to sustain the organization and maintain social harmony (except for *Security-Personal* addressing individual interests in continued employment; Schwartz, 1992). Individuals who value *Tradition* and *Conformity* may thrive in stable environments that demand alignment with managerial direction (Arieli et al., 2020a, 2020b). Valuing stability and compliance in workplaces—as emphasized by *Conservation* work values—is associated with adherence to organizational rules and alignment with management decisions (Arieli et al., 2020b). For instance, during imposed organizational change, employees who prioritize *Conservation* show higher organizational identification (especially *Tradition-Organizational* and *Conformity*; Sverdlik and Oreg, 2015). When, for example, communicating ethical guidelines or implementing organizational change, it can be helpful to tailor the approach depending on whether employees prioritize *Conformity–Interpersonal* (normative expectations and shared values) or *Formal* (detailed, structured policies and role clarity) guiding principles. Both approaches can foster stability and compliance, but they do so through different channels. Additionally, aspects of *Security*, particularly *Security-Organizational*, are relevant when considering safety climate and employees’ motivation to promote safety in the organizational environment to reduce accidents and injuries (Griffin and Curcuruto, 2016).

### The structure of the circular work value theory

Our approach of contextualizing basic values in work contexts ultimately results in an adapted value structure of conflict and compatibility (see Figure 2). This is grounded in the diverse accumulations of previous work value scales which leads to variations in the content and underlying goals and motivational foundations of work values compared to basic values. Additionally, the diverse target levels of the proposed constructs in work settings (individual, job/organizational, societal; Lyons et al., 2010) influence the hypothesized adjustments.

As Figure 2 illustrates, we changed the position of work values in the *Openness to Change* domain, compared to the theory of basic human values. As the definitions of *Self-Direction-Thought* and *Stimulation-Challenge* indicate both facets of intellectual stimulation as using one’s cognitive abilities (*Self-Direction-Thought*) and solve problems/overcome challenges/adapting to new situations (*Stimulation-Challenge*). So, both constructs tend to be theoretically closely related in the motivational circumplex. This results in *Hedonism* being transferred to the border of *Self-Transcendence* work values where *Hedonism-Compatibility* adjoins *Benevolence-Relatedness* and *Hedonism-Pleasure* adjoins *Stimulation-*Var*iety*.

Furthermore, *Self-Direction-Action* is now more closely related to *Achievement-Goal orientation*. As *Self-Direction* work values represent intrapersonal competence, independent of social standards, a close relation to working toward goals and demonstrating initiative and competence is assumed. Here, we postulate *Achievement-Goal orientation* to be located on a Growth—Anxiety-free motivational basis, as this work value is less dependent on external evaluations of performance and action compared to *Achievement-Advancement* (see Schwartz et al., 2012 for comparable differentiations).

We located *Materialism* between *Achievement-Advancement* and *Power-Resources*. The definition of *Materialism* pertains the importance of luxury and wealth through one’s work. This may be highly related to aspects of career progression and recognition according to social standards due to a higher salary in advanced careers. However, importance ascribed to control over material resources in the organization as well can be seen as theoretically related on the motivational continuum, as both focus on material aspects associated with work contexts and goals.

*Power-Dominance* is located between *Power-Resources* and *Status*. As the control over resources and people are both differentiations exhibited in previous literature, this ordering is as well plausible regarding *Status* being more closely related to *Safety-Job*. As Schwartz et al. (2012, p. 666) argued: “Exploiting one’s prestige enables people to control others and to command resources. Protecting one’s prestige entails defending oneself against the threats to one’s security inherent in attacks on one’s public image.” Both, *Status* and *Security-Job*, address securing one’s professional standing while the first focusses on one’s status, prestige and public image, and the latter addresses aspects of job security.

We switched the position of *Tradition* and *Conformity*. As *Conformity-Formal* addresses rules and obligations to enable a structured daily business in organizations, *Security-Organizational* is theoretically closely related due to the importance of stability and safety in the organization and the individual’s work environment. Additionally, *Tradition-Organizational* is more closely related to *Conformity-Interpersonal* as both constructs focus on adhering and complying to non-observable social norms and expectations in organizational surroundings.

*Tradition-Societal* is located on the border to *Sustainability-Environmental* as both constructs exceed organizational boarders. The differentiations address the welfare and continuation of the broader society.

*Sustainability-Social* is closely related to *Equity-Advocacy*. Both focus on the engagement for fairness and justice. However, they differentiate in the target group. The first addresses the societal level while the latter concerns people in one’s work organization.

*Equity-Acceptance* is placed near *Benevolence-Caring.* The first addresses the acceptance of individual differences at work with a broader target group to people whom one encounters at work. The latter focusses on promoting the wellbeing of ingroup members and considering their idiosyncratic needs. Here, *Benevolence-Relatedness* adjoins with the goal of harmonic and trustworthy relationships at work.

### Facilitating the theoretical propositions

In the following, we provide theoretical arguments to support the proposed content and circular structure of work values. We discuss how our work value model addresses the bias in construct conceptualization due to the underdeveloped social dimension of work values (Moors et al., 2017) by drawing parallels to the propositions and controversial influences of neoliberal ideology on workplaces (Bal and Dóci, 2018). Then, we introduce additional theoretical arguments supporting the content and structure of our work value circumplex.

#### Work values and neoliberal ideology

Past research on the theory of basic human values evaluated the associations of basic values to political ideology (Sagiv and Schwartz, 2022). Transferred to workplaces, ideology can be defined as the explicit and deliberate endeavor to create an image of the workplace as it should be and also the lesser known invisible understandings of the social order itself (Bal and Dóci, 2018; Glynos, 2008; Zizek, 1989). An influential but controversial ideology in workplaces and work and organizational psychology in general constitutes neoliberalism (Abrams et al., 2023; Bal and Dóci, 2018; Curran and Hill, 2019; Harvey, 2005; Seubert et al., 2023). As a political-economic ideology, human wellbeing is to be maximized by economic freedom in societies (Fine and Saad-Filho, 2017; Harvey, 2005). How neoliberal ideology can be conceptualized and how it may influences workplace behavior is done comprehensively elsewhere (see Bal and Dóci, 2018). We aim at referring our work value model to the basic motivational foundations of neoliberalism given by Bal and Dóci (2018). We examine how our model helps to expand the perspectives on what individuals expect from work, and therefore potentially constitutes a less biased, cross-culturally generalizable conceptualization of work values.

Thus, we focus on the fantasmatic logic of neoliberalism to differentiate potential interconnections in the work value circumplex on a more abstract, conceptual level (Bal and Dóci, 2018; Glynos, 2008). In contrast, other logics of neoliberal ideology also relate to what individuals value at work and which guiding principles influence behavior and decision-making in organizations. The political logic defines core rules and norms (e.g., individualism and competition) and shapes how political discourse is constructed under neoliberalism (Glynos, 2008). The social logic represents the influences on employees’ working lives in a more tangible way, addressing practices such as control and monitoring as manifestations of ideological motivations and the enactment of norms (Morgan, 2015). Our focus, however, lies on the fantasmatic logic, as it explains why certain practices persist, why they are appealing to individuals, and how they motivate continuation of neoliberalism and its influence as desirable beliefs on workplaces (Glynos, 2008, 2011). At this level, definitional parallels emerge with our understanding of work values as broad and fundamental, desirable goals that guide decision-making and behavior in organizations.

The first fantasy of neoliberalism is the freedom of individuals. As Bal and Dóci (2018, p. 5) illustrated, “the center of the freedom fantasy is the agentic and free individual who can take care of her/himself [and] who is in no need of the state’s, the organization’s or any authority’s protection.” Neoliberalism provides the fantasy to liberate employees from bureaucratic organizations and the paternalistic influence of collectives. Hence, employees have the opportunity to pursuit their own interests and strive for self-fulfillment on a competitive market (Bal and Dóci, 2018). Individuals are responsible for their own employment and employability (Bal and Jansen, 2016). Here, relations to personal focused work values, especially *Openness to Change* constructs as *Self-Direction* or *Stimulation* become apparent. These are opposed to organizational or cultural influences on individuals in *Conservation* work values of *Tradition* and *Conformity*. Additionally, the competitiveness and the individualization of employability, personal development and success (as indicators for the *Self-Enhancement* dimension) conflicts with *Self-Transcendence* work values.

Meritocracy and social Darwinism form the second fantasy of neoliberal ideology (Bal and Dóci, 2018). Meritocracy, the concept that rewards in society and the workplace should be based on merit and talent, is often depicted as the belief that success stems from individual qualities like willpower and hard work rather than inherited advantages (Ayers and Saad-Filho, 2015; Bourdieu, 1986; Castilla and Benard, 2010). However, this idealized notion disregards the unequal distribution of resources and privileges among the elites due to structural power differences like social class and ethnicity (Burke, 2013; Littler, 2013). In relation to social Darwinism, neoliberalism stresses the natural selection of the strong and capable surviving in a competitive market. Competition is seen as fair as everyone has the same chances to make use of their freedom and strive for success. As a result, the society distinguishes between those who “succeed” and those who “loose.” Thus, social injustice and exploitation is justifiable, as success is based on merit, talent, and the strength and capability in a competitive market (Bal and Dóci, 2018). Transferring these assumptions to our work value model, the conflicting guiding principles of *Self-Enhancement* and *Self-Transcendence* become clear. While the first emphasizes power, authority, influence, and success at work through one’ own competitive advancements, the latter focusses on the meaningfulness of positive social relationships and the possibility to contribute to an equal and sustainable society through work.

The last motivational foundation of neoliberalism is the belief in growth and progress. Influenced by growth economies on a societal level, growing in status or personally is seen as desirable and inherently good on an individual level (Bal and Dóci, 2018). Hence, personal focused work values, especially the dimension of *Self-Enhancement*, are key guiding principles to be aligned with this conviction. Increasing one’s own market value based on external assessments is the ultimate goal. Again, the conflict to *Self-Transcendence* work values can be emphasized by following illustration: “if it is the individual’s striving for personal growth and progress that makes society as a whole well-functioning, then it is entirely legitimate and desirable that individuals care primarily about their own interests, strive to outcompete others and regard others instrumental in this process.“(Bal and Dóci, 2018, p. 7). Accordingly, the focus on the individuals’ own interests is conflicted with broad group goals as illustrated by work values of *Conservation* opposed to *Openness to Change*.

In conclusion, our proposed work value circumplex can be related to the fantasmatic motivational foundations of neoliberalism. Work values in the personal-focused dimensions of *Openness to Change* and especially *Self-Enhancement* are theoretically associated with neoliberal propositions. The underlying conflict of the work value circumplex is illustrated by the representation of oppositions and critiques in neoliberal fantasies within social-focused work value dimension such as *Conservation* and *Self-Transcendence* with a focus on collective needs and the welfare of others. Compared to past research on work values, a relative underrepresentation of work values in the social-focused dimension is apparent (Busque-Carrier et al., 2022a; Moors et al., 2017). Additionally, the constructs reviewed for the IWVS are predominantly located on a personal-focused dimension. Thus, the range of construct content in past studies may be too restricted, as indicated by a disproportionate alignment with neoliberal assumptions and a bias in work value conceptualizations (Moors et al., 2017). We next present additional arguments orbiting these central propositions.

#### Expanding the framework: theoretical support for the CWVT

For providing additional examples to foster the content and structure of our work value model, we summarized further theoretical arguments in Table 3. Here, theories which depict the relevance of specific work values to explain organizational behavior (e.g., CSR, Meaning of Work, PERMA+4) and support the circumplex nature of conflicting and complementing work values (e.g., ethical leadership, goal-oriented motivation) are presented. We do not aim at discussing causal relationships between values and behavior, as the association is much more complex than bivariate relations (Maio et al., 2020; Sagiv and Roccas, 2021). Additionally, this list is not exhaustive. Our goal is to provide exemplifying support for the content validity of our theory and the circumplex nature of work value associations.

**Table 3:** Additional theoretical arguments in relation to the proposed CWVT.

Theory	Content	Exemplary relations to CWVT
Corporate social responsibility (CSR) (Glavas, 2016; Paruzel et al., 2021)	Other-regarding values moderating the effect of Macro-CSR (organizational level) on Micro-CSR outcomes (individual level) CSR Social-dimension differentiated into people-society and people-employee	Social vs. personal focus, equity and sustainability
Ethical leadership (Bass and Steidlmeier, 1999)	Moral intention (egoism vs. altruism) and moral consequences (benefits and costs for self vs. others) Transactional leadership models are grounded in a worldview of self-interest Authentic transformational leadership depicts a self that is connected to social environment (importance of others welfare) Instead of imposing ethical norms and behavioral ideals, they should be freely embraced. Authentic inner commitment should be the basis of motivation rather than coercion. Encouraging questioning and creativity is crucial.	Social vs. personal focus, openness to change vs. conservation, self-transcendence vs. self-enhancement
Economic ideology and national culture (Inglehart and Baker, 2000; Ralston et al., 2008)	Industrialization was linked with an emphasis on economic growth, physical and economic security Post-industrialization placed increasing emphasis on quality-of-life, environmental protection, and self-expression (postmaterialist and postmodern values)	Growth - anxiety-free vs. self-protection—anxiety-avoidance, openness to change vs. conservation, self-transcendence vs. self-enhancement
Individual wellbeing conceptualized by the PERMA+4 model (Donaldson et al., 2022)	Building blocks for wellbeing: Positive emotions, engagement, relationships, meaning, accomplishment Additional building blocks for work-related wellbeing and performance: Physical health, Mindset, Work Environment, Economic security	Hedonism, self-direction, benevolence, sustainability, achievement, security
Workplace fun (Tews et al., 2014)	Role of workplace fun (activities providing amusement, enjoyment or pleasure) as theoretical advancement in explaining turnover intention	Hedonism
Need theories (Busque-Carrier et al., 2022b; Deci and Ryan, 2000; Steers et al., 2004)	Self-Determination-Theory (Autonomy, Relatedness, Competence), with a focus on growth-oriented activity McClelland’s need theory additionally focused on needs for achievement (competition with a standard of excellence) and power (control over one’s environment) intrinsic and social work values are positively related to psychological need satisfaction (PNS) at work and negatively to psychological need frustration (PNF) at work, whereas extrinsic and status work values are positively associated to PNF and negatively to PNS	Growth - anxiety-free vs. self-protection—anxiety-avoidance, self-enhancement vs. self-transcendence, openness to change vs. conservation, Self-direction, benevolence, achievement-goal orientation, power
Goal oriented motivation / Goal setting theory (Butera et al., 2024; Elliot and McGregor, 2001)	Learning goal orientation seeks to increase competence through skill and task mastery Performance goal orientation focus on the result of demonstrating competence through showing adequate or excellent performance Differentiated in approach and avoidance goal orientation	Growth - anxiety-free vs. self-protection—anxiety-avoidance, achievement vs. self-direction, achievement-advancement vs. achievement-goal orientation, stimulation-challenge
Materialism (Kasser, 2016)	Materialism orients people toward superficial satisfactions and conflicts with caring about the broader world, one’s family, and/or religious pursuits (integration in theory of basic human values as a *Self-Enhancement* work value)	Materialism vs. self-transcendence
Characteristics of precarious and decent work (Allan et al., 2021; Seubert et al., 2021)	Precarious work: Job/employment insecurity, workplace uncertainty; lack of psychosocial safety, social rejection, discrimination; lack of need satisfaction; poverty wage Decent work: Job/planning security and living wage; social networks with communication and cooperation; status and recognition; meaning in work	Status, security, sustainability, equity, benevolence, self-direction
Bureaucratic organizations (Inglehart and Baker, 2000; Putnam et al., 1994)	Horizontal, locally controlled organizations are conducive to interpersonal trust whereas rule by large, hierarchical, centralized bureaucracies seems to corrode interpersonal trust.	Conservation vs. openness to change, conformity
Organizational culture (Schein, 2017)	Three levels of organizational culture influencing individual behavior: Artefacts as observable structures and processes, espoused beliefs and values as less observable norms and behavioral rules, underlying assumptions as unconscious beliefs and thoughts	Conformity, tradition
Cultural values (Schwartz, 1999)	National differences in cultural values and norms about work influence MW	Tradition-societal
Religion and spirituality at work (Dik et al., 2024)	Influence of religion and spirituality on organizational outcomes and especially the conceptualization of MW	Tradition-societal

As discussed above, referring employees’ work values to their work environment is a fundamental perspective in conducting research on PO fit (Barrick and Parks-Leduc, 2019). As our developed theory is situated on the person-level, we want to further explore its referability to the organization-level. De Clercq et al. (2008) argued for the TBHV to be useful perspective in determining fit, as associations of conflict and compatibility are directly displayed in a comprehensive theoretical circularity. One example supporting this perspective is the relation of basic human values to the OCP (O’Reilly et al., 1991) by Borg et al. (2011). We want to build on these findings and relate the CWVT to additional empirically supported theory-based instruments for assessing organizational culture (Puppatz et al., 2017). Hence, we refer to the Denison Organizational Culture Survey (DOCS; Denison et al., 2014), the revised OCP (Sarros et al., 2005). The relations are displayed in Table 4.

**Table 4:** Organizational culture dimensions and relations to the CWVT.

Organizational culture model	Dimensions and definitions	Relations to CWVT
DOCS (Denison et al., 2014)	Involvement—empowerment: individuals have the authority, initiative, and ability to manage their own work. This creates a sense of ownership and responsibility toward the organization.	Self-direction, achievement-goal orientation
	Involvement—team orientation: value is placed on working cooperatively toward common goals for which all employees feel mutually accountable. The organization relies on team effort to get work done.	Benevolence
	Involvement—capability development: the organization continually invests in the development of employees’ skills in order to stay competitive and meet ongoing business needs.	Self-direction, achievement
	Consistency—core values: members of the organization share a set of values which create a sense of identity and a clear set of expectations.	Tradition-organizational, conformity-informal
	Consistency—agreement: members of the organization are able to reach agreement on critical issues. This includes both the underlying level of agreement and the ability to reconcile differences when they occur.	Benevolence, equity
	Consistency—coordination and integration: different functions and units of the organization are able to work together well to achieve common goals. Organizational boundaries do not interfere with getting work done.	Benevolence
	Adaptability—creating change: the organization is able to create adaptive ways to meet changing needs. It can read the business environment, react quickly to current trends, and anticipate future changes.	Stimulation
	Adaptability—customer focus: the organization understands and reacts to their customers and anticipates their future needs. It reflects the degree to which the organization is driven by a concern to satisfy their customers.	–
	Adaptability—organizational learning: the organization receives, translates, and interprets signals from the environment into opportunities for encouraging innovation, gaining knowledge, and developing capabilities.	Stimulation, self-direction
	Mission—strategic direction and intent: clear strategic intentions convey the organization’s purpose and make it clear how everyone can contribute and “make their mark” on the industry.	Tradition-organization, conformity
	Mission—goals and objectives: a clear set of goals and objectives can be linked to the mission, vision, and strategy, and provide everyone with a clear direction in their work.	Achievement
	Mission—vision: the organization has a shared view of a desired future state. It embodies core values and captures the hearts and minds of the organization’s people, while providing guidance and direction.	Tradition-organizational, conformity, sustainability, equity
Revised OCP (Sarros et al., 2005)	Competitiveness: achievement orientation, An emphasis on quality, Being distinctive—being different from others, Being competitive	Achievement, power, status
	Social responsibility: being reflective, Having a good reputation, Being socially responsible, Having a clear guiding philosophy	Sustainability, equity, tradition-societal
	Supportiveness: being team oriented, Sharing information freely, Being people oriented, Collaboration	Benevolence, equity
	Innovation: being innovative, Quick to take advantage of opportunities, Risk taking, Taking individual responsibility	Self-direction, stimulation
	Emphasis on rewards: fairness, opportunities for professional growth, High pay for good performance, Praise for good performance	Equity, self-direction, achievement, materialism, power, status
	Performance orientation: having high expectations for performance, Enthusiasm for the job, Being results oriented, Being highly organized	Achievement, hedonism, power, conformity
	Stability: stability, Being calm, Security of employment	Tradition, conformity, security

To support the transferability of our hypothesized structure of conflict and compatibility to work contexts we elaborate on two examples from related fields of work and organizational psychology mentioned in Tables 3, 4—meaning of work and organizational culture. First, a model of meaning of work (MW) developed by Rosso et al. (2010) through an integrative review hypothesizes four major pathways to meaningful work (self-connection, individuation, contribution, unification). These pathways are positioned between the opposing motivations of agency (drive to differentiate, master and create) and communion (drive to contact, attach and unite) and the target of one’ actions (self or others). Spanning these four distinctions, different actions and behaviors can guide individuals to meaningful work. Here are parallels to the continuum hypothesized by the CWVT. The opposing motivational foundations in social and personal work values (see Figure 2) align with the hypothesized differentiations of pathways to meaningful work. For example, individuation (in the agency-self quadrant) emphasizes control, autonomy and competence of individuals, while on the other end, unification (in the communion-others quadrant) aims at bringing individuals into harmony with other beings or principles (Rosso et al., 2010). These differentiations align with our hypothesized conflict between *Openness to Change* / *Self-Enhancement* work values (especially *Achievement*, *Stimulation* and *Self-Direction*) and *Conservation* / *Self-Transcendence* (especially *Tradition*, *Conformity*, *Equity, Sustainability and Benevolence*).

Second, from an organizational culture perspective, the Competing Values Framework (CVF, Quinn and Rohrbaugh, 1983) represents competing strategic core values addressing different aspects relevant for an organizations’ performance. The CVF was used to establish links to organizational effectiveness (Hartnell et al., 2011) and organizing various domain-specific organizational climates (Beus et al., 2020). Four culture types are distinguished in the CVF: clan (focus on attachment, collaboration, affiliation), adhocracy (focus on growth, stimulation, variety and autonomy), market (focus on competition, competence and achievement) and hierarchy (focus on routinization, formalization and consistency). The four distinct cultural types are referable to the higher-order work values as proposed by the CWVT: *Self-Transcendence* work values relate to the clan-type, *Openness to Change* to adhocracy, *Self-Enhancement* to market-culture and *Conservation* to hierarchy. Here, the conflicting structure of opposed beliefs and guiding principles becomes apparent on an organizational level. Structuring domain-specific organizational climates in this framework, Beus et al. (2020) highlight aspects of conflict and compatibility among these shared perceptions of organizational practices. In line with the CWVT, climates of support, justice/fairness, teamwork, trust, cooperation and caring were identified to relate to the clan culture-type (*Self-Transcendence*). Adhocracy incorporates climates of innovation, empowerment, autonomy, creativity and risk-taking which are in line with the work values of *Openness to Change*. *Self-Enhancement* work values are aligned with climates of achievement, performance, recognition, goal orientation and work pressure, as manifestations of the market culture-type. The hierarchy culture-type differentiates climates of structure, role clarity, safety, control and ethical climate, which relates to *Conservation* work values (where an ethical climate might be already on the transition to *Sustainability* as an adjacent work value).

Overall, these two examples align the assumptions of conflict and compatibility of the CWVT with previous research in the field of work and organizational psychology. In the following, we will conclude our theorizing with a discussion of our arguments.

## Discussion

The aim of this paper was to develop conceptual clarity of what work values are and what not, how they can be integrated into a theoretical framework and how this framework might help us to illuminate blind spots and biases in past approaches. Building on the IWVS (Busque-Carrier et al., 2022a) we illuminated biases in past research with a focus on lists of constructs lacking an unifying theoretical framework. In the following, we will discuss and summarize the four dominant contributions we believe our paper provides.

We presented a work value definition which fits the definition of basic personal values (Sagiv and Schwartz, 2022). Here, we were guided by the premise that work values are a contextualization of individuals’ basic values. Discussing why these definitional aspects matter in the realm of organizations provides a meaningful foundation for future work value studies tackling the issue of construct proliferation. A shared conceptualization of work values referrable to the definition of basic values enables research to consider the advancements in basic value literature and establish contextual sensitivity of new findings for theorizing in work and organizational psychology.

Building on this, our theory integrates past scale developments reviewed by the IWVS into a single comprehensive framework, namely the extensively researched and cross-culturally validated theory of basic human values. By providing a unifying theoretical framework for work value research we hope to foster theoretical considerations in future studies. The elaboration of the theory of basic human values and its contextual advancements in organizational settings enable researchers to ask more amplified questions due to the large background on behavioral implications of basic value research (Arieli et al., 2020b; Sagiv and Roccas, 2021). The contextual sensitivity of the theory potentially enhances the precision and relevance of research questions in work settings. For example, researchers could use the work value model to study the effects of value congruence in identity leadership theory and team settings. Specific questions might be asked to address which work values in the circumplex might be more important when examining congruence effects in organizational behavior. Another potential research array may be the role of self-affirmation theory and its effect on wellbeing given different motivational foundations in the work value circumplex.

Our discussion of potential behavioral implications for the different work values can enhance the practical relevance of the CWVT given their desirable outcomes for organizations (e.g., green employee behavior, compliance and safety behavior, pursuit of success, creativity and flexibility). Past studies support the possibilities for volunteer value change utilizing the value conceptualizations of the TBHV (Russo et al., 2022). This highlights the usefulness of work value considerations in for example organizational socialization or personnel development. Moreover, identifying employees work values and relate them to associated behaviors can help individuals to act on their work values and foster their wellbeing (Bojanowska et al., 2022; Russo-Netzer and Atad, 2024). This may be especially relevant for career counseling or coaching. Here, the presented behaviors and favored organizational circumstances can provide initial directions for employees’ to align their work values with their work behavior and preferences. Moreover, leadership and team development might benefit from highlighting the prevalent work values in teams and especially the shared values across team members and their leaders. This paves the way for interventions based on the identity leadership perspective (Haslam et al., 2022) to develop a shared sense of social identity in teams (Haslam et al., 2023).

Our review of neoliberal ideology in relation to our work value theory displayed the conflict of social vs. personal focused work values. Past scales predominantly focused on work values attributable to the personal dimension (Busque-Carrier et al., 2022a) with an underdevelopment of social work values (Moors et al., 2017). The former are conceptually more aligned with the propositions given by the fantasmatic logic of neoliberalism. As a result, perspectives on work values might be too narrow and lack cross-cultural generalizability, as national culture or economic ideology can influence personal value systems (Ralston et al., 2008, 2011; Schwartz, 1999). The universal approach of the theory of basic human values addresses this gap (Schwartz, 1992). Thus, we believe that our model provides a more comprehensive and cross-culturally sensitive picture of relevant work values exceeding neoliberal restrictions. Based on the universality of Schwartz’ theory, this enlarges the perspectives and possibilities for theorizing as for example cross-cultural and out-of-the box hypothesis might be considered as well as more macro-level influences and guiding principles like *Conformity* or *Tradition* (Anseel et al., 2018).

We are aware of the controversial discussions around the topic of ideology and its influences on our work (Anseel et al., 2018; Rudolph and Zacher, 2018). Nonetheless, we aimed to reinforce the circumplex model of work values and uncover blind spots in current literature. Past research identified this motivational continuum based on empirical data (Albrecht et al., 2020; Borg et al., 2019; Lyons et al., 2010; Schneider et al., 2024). As our theoretical associations illustrate, conceptualizing work values in a circumplex with conflicting and complementing basic motivational goals and guiding principles can provide useful extensions not just for the internal structures of work values themselves, but also in relation to other theories. For example, the theoretical foundation of a work value circumplex expands perspectives on relations to CSR, meaning of work, religion and spirituality in work contexts, differentiations of learning and performance goal orientation as well as the supplementary fit between person and environment (conflict or compatibility of individuals’ work values to organizational values along the motivational continuum; Kristof, 1996).

Given the different levels of abstraction, one might choose the level most useful for their research question and variable of interest. Table 3 illustrates that even the most abstract dimensions could provide useful insights in explaining relations (*Growth - Anxiety-free* and *Self-Protection—Anxiety-avoidance*). Hence, we refer to the discussion around narrower and broader variables given in the bandwidth-fidelity-dilemma (Ones and Viswesvaran, 1996; Salgado, 2017) and encourage the use of more abstract work values when interested in more abstract outcomes.

### Limitations and future research

In the following, we will discuss pathways for future research and address limitations of our here presented theorizing.

#### Empirically evaluating differentiations

Future studies should aim to provide further support for the proposed differentiation of work values, including assessments of the incremental validity of the specified sub-constructs. In light of the theoretical principle of parsimony, it is essential to evaluate the empirical distinctiveness of these dimensions to ensure that practical recommendations remain meaningful and actionable (Aguinis and Cronin, 2022).

In this context, an important theoretical limitation must be acknowledged: the CWVT introduces additional assumptions and complexity, making it a less parsimonious and a less generalizable framework compared to the TBHV. Therefore, further empirical research is needed to determine whether the CWVT offers added explanatory value for work-related phenomena beyond what the TBHV already accounts for (see hypothesized behavioral implications above).

#### Generalizability of the CWVT to various cultures with methodological triangulations

Given the relative lack of previous work value constructs in the social-focused dimension, their validity and cross-cultural replicability needs to be considered. We aimed at providing more comprehensive constructs compared to former work value scales. This was inspired by Schwartz’ work on cross-cultural and universal basic value structures which was replicated in many different societies on large data sets (Schwartz, 1994; Schwartz et al., 2012; Schwartz and Cieciuch, 2022). However, we encourage a critical examination of our unifying work value model. Developing appropriate instruments to empirically corroborate the model is required by data collections and studies in various cultures. Thus, a comprehensive theory driven scale development approach using state-of-the-art validation practices (Cortina et al., 2020) should be the center of future work value research.

Thus, scale validation approaches should build on past (work) value scales (Albrecht et al., 2020; Busque-Carrier et al., 2022a; Consiglio et al., 2017; Schneider et al., 2024; Schwartz and Cieciuch, 2022) to align work value assessment with the circularity assumption of the CWVT. Example items could address contextualized wordings of the PVQ-RR (Schwartz and Cieciuch, 2022), where respondents compare a described person to themselves and indicate, how similar the described person is to them (e.g., “It is important to this person to have the freedom to choose what they do at work.” for *Self-Direction-Action*; “It is important to this person to develop their own ideas at work, regardless of what others think.” for *Self-Direction-Thought*). Initial content validation should take place by incorporating expert feedback from researchers on how well the items represent the derived work value definitions in Table 2. Building on this item pool, cognitive interviews with employees can be useful to investigate the understandability of item contents and gain a first impression of how employees rate the discussed guiding principles. With sufficiently large sample sizes, an initial test of the factorial structure can be conducted. Translating the items and testing them in other languages should make the CWVT, its generalizability and applicability in other countries more accessible (especially replicating the circular structure and the measurement model through measurement invariance testing).

Building on this, initial behavioral implications can be researched, as an assessment of the CWVT now allows researchers to analyze work values’ associations to behavioral outcomes. For example, self-rated, other-rated behaviors or decision-making in ill-defined problems (Mumford et al., 2002) can help researchers to understand the practical utility of here discussed work values.

A key limitation of our approach lies in the reliance on predominantly quantitatively derived and researched frameworks (Busque-Carrier et al., 2022a; Schneider et al., 2024; Schwartz and Cieciuch, 2022). While these models are grounded in cross-culturally validated theories, they may still constrain the range of perspectives captured by the developed CWVT. To address this, future research could incorporate additional data sources—such as exploratory analyses of written or spoken language using large language models and natural language processing (e.g., Ponizovskiy et al., 2020; Tonidandel et al., 2022)—to gain deeper insights into the guiding principles of employees.

#### Workplace specificities of behavioral implications

Another important alley for future research are the implications of work values for behaviors in organizations (Maio et al., 2020). Sagiv and Roccas (2021) identified processes and variables which may influence the value-behavior association. For example, the factor of “control” represented by external *Conformity* values, social norms or cultural tightness/looseness should be specified for organizations. Here, the job design might as well be a contextual moderator. Job autonomy, leadership, career advancement possibilities or social support by colleagues can potentially moderate the extent to which work values are translatable into organizational behavior (Barrick and Parks-Leduc, 2019). Additional theorizing is required to develop hypotheses on contextualizing the variables identified to influence the value-behavior nexus (Arieli et al., 2020a; Sagiv and Roccas, 2021).

The behavioral implications of each higher-order work value—and the associated differentiations—warrant extensive empirical investigation, taking into account the process variables outlined above that may moderate these relationships. Some of these implications are grounded in empirical studies of work values; others are theoretical hypotheses based on content and conceptual similarities in value–behavior linkages. Consequently, future research should undertake a rigorous, data-driven evaluation of the CWVT and its nomological network.

#### Aligning the CWVT with fit-research

We believe that the proposed model could advance the literature on PO-Fit. Due to mixed results in fit studies (Van Vianen, 2018), methodological developments in assessing mis−/fit (Yao and Ma, 2023) and proliferation of organizational value theories (De Clercq et al., 2008) the circular work value theory could provide meaningful advancements in studying value congruence. This stems from the basic proposition of our theory, as conflict and compatibility are inherent to the hypothesized structure based on an elaborated and extensively researched framework of individuals’ basic values. Thus, using this comprehensive theory to assess in−/congruence and consider the influence of different fit attributes (e.g., work values with different motivational foundations) could strengthen the theoretical basis of future PO-fit research (Kristof-Brown et al., 2023).

As shown in Table 4, the CWVT demonstrates meaningful parallels with established organizational culture models, providing a useful starting point for exploring hypothesized patterns of conflict and compatibility. In particular, the dimension emphasis on rewards in the revised OCP (Sarros et al., 2005) may be of interest, as it can be conceptually linked to the work values of *Self-Transcendence* and *Self-Enhancement*. Analyzing individual work value profiles may offer deeper insights into patterns of fit and misfit, especially in the context of such inconsistent definitions from a personal work value standpoint (Arieli et al., 2020b).

Some limitations, however, must be acknowledged. For example, not all dimensions of the DOCS could be clearly mapped onto our hypothesized circular continuum—Customer Focus being a case in point. This discrepancy may stem from differing levels of abstraction, as not all employees—or even entire organizations such as public sector agencies—have direct customer contact (e.g., HR departments). In such cases, emphasizing customer needs may be too context-specific and misaligned with the broader definition of work values employed in this study. Furthermore, even though our discussion of the CVF related to conflict and compatibility aligned with the assumptions of the CWVT, some limitations must be acknowledged. Past meta-analytical findings provide limited support of the nomological associations and the hypothesized internal structure of the CVF (Hartnell et al., 2011). Thus, Hartnell et al. (2011) suggest to not view the CVF as distinct and mutually exclusive types of cultures, but as cultural profiles and patterns among associated values. Supporting this view, the CWVT can provide a perspective on organizational culture where the theoretical bandwidth is captured by various configurations of beliefs and work values, and not as distinctive types. This should be considered in future fit research when aligning employees work values to organizational culture profiles.

This observation also points to a broader issue: the need for continuous evaluation of the relevance and comprehensiveness of work values. As Kristof-Brown et al. (2023) emphasize, value profiles may require regular updating due to societal developments. Recent extensions of the TBHV support this view—for instance, *Sustainability* (Albrecht et al., 2020) and *Hedonism-Compatibility* (Consiglio et al., 2017) have been introduced as contextualized expressions of *Universalism* and *Hedonism*. These developments highlight the importance of remaining attuned to societal change and its influence on the evolving salience of work values. Nevertheless, evolving guiding principles at work should continue to align with the definition of work values as beliefs and broad goals that help individuals cope with the fundamental needs of human existence—including biological needs, the requirements of coordinated social interaction, and the survival and welfare of groups. When updating personal work value theories, it is important to avoid reverting to overly specific or potentially biased value lists, thereby minimizing the risk of construct proliferation.

## Conclusion

In conclusion, this paper proposes a theoretical framework for work value research by leveraging the widely acknowledged and unifying assumptions of the theory of basic human values. We addressed three major gaps in the literature: establishing a theoretical foundation for a more sound basis in work value research, offering diverse perspectives on guiding principles at work to enhance the richness of hypotheses formulation and cross-cultural generalizability, and providing clarity on the essential work values to assess amidst prevalent construct proliferation and ambiguity. Our theoretical approach draws on insights from psychology and related disciplines. We believe this foundation will significantly benefit future studies in work values and inspire critical evaluation of our proposed theory.

## Data Availability

The original contributions presented in the study are included in the article/supplementary material, further inquiries can be directed to the corresponding authors.

## Footnotes

^1^For clearer conceptualization of higher-order work value dimensions and to underline our approach of contextualizing the theory of basic human values, we continue to refer to *Self-Transcendence*, *Self-Enhancement*, *Openness to Change* and *Conservation* when discussing work value dimensions. Additionally, we referred our work value labels to the theory of basic human values and the contextualization of Consiglio et al. (2017) to avoid contributing to jingle-jangle fallacy (Flake and Fried, 2020).

^2^Schwartz et al. (2012) integrated an additional value of *Humility* between the dimensions of *Conservation* and *Self-Transcendence*. Past contextualizations of the TBHV and the integrative work for the IWVS (Busque-Carrier et al., 2022a) provide no evidence of such a value in work contexts (research on humility in organizations typically conceptualizes humbleness as a personality trait or specific leadership behavior, rather than as a desirable belief that serves as a guiding principle in employees’ lives; e.g., Chandler et al., 2023; Lee et al., 2019). Hence, we consider the higher-order work value of *Self-Enhancement* and especially *Achievement-Advancement* and *Materialism* to be the opposite of a potential *Humility* work value, as the connectivity to existing work value literature is more apparent. This is supported by considering the definition of the *Humility* basic value as “recognizing one’s insignificance in the larger scheme of things” and the conflicting goal of luxury, material goods and recognition according to social standards as well as influence on other individuals in the Self-Enhancement dimension. Additionally, empirical studies indicate high negative associations between *Humility* and *Materialism* (Pilch and Górnik-Durose, 2016) supporting the conflict between these opposing guiding principles.
